# Frequency of parasitic infections in *Arachishypogaea L* (groundnuts), *Citrulluslanatus* seeds (watermelon seeds), and *Ziziphusspina-christi* (nabag) sold by street vendors in Khartoum State, Sudan: a cross-sectional study

**DOI:** 10.12688/f1000research.53682.3

**Published:** 2021-12-15

**Authors:** Arwa Suleiman Mohammed, Ahmed Abd Alla, Ahmed Galander, Tayseer Elfaki, Ahmed Ibrahim Hashim, Hisham N. Altayb

**Affiliations:** 1 Parasitology and Medical Entomology, Sudan University of Science and Technology, Khartoum, Khartoum, 11111, Sudan; 2 Microbiology, Sudan University of Science and Technology, Khartoum, Khartoum, 11111, Sudan; 3 Biochemistry, College of science, King Abdulaziz University,, King Abdulaziz University, Gedda, Gedda, Saudi Arabia

**Keywords:** Arachis hypogaea L, Groundnuts, Citrullus lanatus, Nabag, Tasali

## Abstract

**Background**: Plant products, including seeds are an important source of vitamins, minerals, proteins, and energy. This study aimed to assess parasitic contaminations in roasted groundnuts,

nabag, and tasali (watermelon seeds) sold by street vendors in Khartoum State, Sudan.

**Methods:** The frequency of parasitic contaminations among all crop products was detected by washing the plants with saline, and then conducting an examination using a formal ether

concentration technique (FECT), followed by a saturated sugar floatation technique.

**Results: **The detected parasites belonged to two species: *Entamoeba histolytica* (33.3%) and *Giardia lamblia* (15.6%). No helminthic parasites were detected. Mixed contamination of the mentioned parasites was also observed (11.1%). The most contaminated crop was nabag, followed by groundnut, and finally tasali.

**Conclusion:** No relation was established between the positivity of samples for parasites and crop type, Khartoum State city, or  seller sex. FECT was more sensitive than the saturated sugar floatation technique as a detection method.

## Introduction

Intestinal parasitic infections can be transmitted orally through the ingestion of infective agents from infected food, water, or contaminated hands ^1, 2^. Food contamination and food borne parasitic diseases frequently occur globally. These are estimated to amount to 23.2 million cases and 45,927 deaths annually ^3^. Fresh fruits and vegetables could be a source of dissemination of foodborne parasitic diseases ^4, 5^. A study in Ghana revealed *Ascaris lumbricoides* (roundworm), *Ancylostoma duodenale* (nematode), *Necator americanus* (hookworm), and *Strongyloides stercoralis* (threadworm) contaminations in tiger nuts ^6^. Another study on tiger nuts reported other contaminants such as animal droppings, fungi toxins and bacteria ^7^. Groundnuts, *Ziziphus spina-christi* (nabag), and watermelon seeds (tasali) are widely consumed in Africa and the Middle East ^8^. Nabag and tasali are widely eaten in Sudan.

These crops are natural sources of carbohydrates, proteins, fats, -iron, calcium, ascorbic acid, thiamine, riboflavin, and niacin ^9– 12^. Groundnuts in Sudan are mainly used for oil extraction, but can be eaten as a snack: raw, or after roasting without its external cortex envelop ^13^. Nabag is eaten after the sweet pulp of the fruit is dried to produce fine flour ^14^. The flour is placed in small metal cups and steamed. Dried pulp flour and water are also mixed with sesame and shaped into small balls ^15^. Fruit pulp prepared in these two ways can be consumed either immediately or stored for future use. In addition to groundnuts, nabag, and tasali ^16^, many crop products represent an important and for some, the only source of income in Sudan ^15^.

Intestinal parasitic infections are very common worldwide. They are often not diagnosed and hence not treated, leading to harmful effects which can be lethal in some cases. Food contamination may occur when food is prepared, stored, or handled; this is a common phenomenon in public places like the streets ^15^. Identifying parasitic contamination will help fight these infections, because knowing contamination rates allows to take the necessary preventive measures. The objective of this study was to identify parasitic contamination rates in *Arachis hypogaea L* (groundnuts), *Citrullus lanatus* seeds (watermelon seeds), and *Ziziphus spina-christi* (nabag) sold by street vendors in Khartoum State, in Sudan.

## Method

### Study design

This descriptive cross-sectional study was conducted between July and October 2019 in Khartoum State, Sudan. The study included the Khartoum cities Khartoum, Bahri, and Omdurman.

### Ethical considerations

Ethical approval for this study was received from the Sudan University of Science and Technology’s Committee of Medical Laboratory Science, with the ethical approval number (MLS – IEC – 03 – 18). All participants in this study issued written informed consent for participation and data publication; for participants under 18 years old, consent was obtained from their guardians.

### Sampling

The study was conducted on street vendors in Khartoum State, sampling groundnuts, nabag, and tasali. In total, 69.8 g of nabag, 50 g of tasali, and 69.9 g of groundnuts were purchased from 15 sellers in Sudan’s Khartoum state (five sellers from each city of interest, i.e. Bahri, Khartoum, and Omdurman), using a cluster random sampling technique, for a total of 45 samples (15 samples from each crop product type). The items were brought to the laboratory and tested under a microscope for parasitic agents. Sellers’ location, gender, and age group were observed.

### Sample processing

Each product purchased from the same seller was put separately in clean, dry glass bottles after labelling, which were thenfilled up to the surface with distilled water. Bottles were left for one hour, and then the nabag, tasali, and groundnut were removed from the liquid using a plastic sieve; the washes were collected and then examined using a formal ether concentration technique (FECT) and saturated sugar floatation technique.

### Formal ether concentration technique

Nabag and tasali washes were added to 4 ml of 10% volume per volume formal saline contained in a conical centrifuge tube. The contents were well-mixed by centrifuging for 20 seconds. After centrifugation four layers of ether, plant debris, formal saline, and deposit were discarded using a sterile plastic Pastier pipette. The deposits of sieved wash liquid were examined under a microscope using 10× and 40× magnification, to detect parasitic agents such as cysts,trophozoite larva, helminth eggs, and species *G. lamblia, E. histolytica, A. lumbricoides, Trichuris trichiura, A. doudenale, N. americanus* and *S. stercoralis*) ^17^.

### Saturated sugar solution floatation technique

Approximately 1 ml of each previously prepared crop wash was put into a glass tube; afterthat, the floatation solution (saturated sugar) was added gradually until a convex surface was formed on the top of the glass tube, which was covered with a glass cover. The tube was left for 15–30 min, after which the glass cover was removed; the solution was put on a microscope slide, and examined under the microscope to detect parasitic agents ^17^.

### Statistical analysis

The statistical package for social science (SPSS, IBM) version 20 program, was used for data analysis. Statistical tests were performed at a 5% significance level ( *P* < 0.05) and a confidence interval (CI) of 95%. The measured frequencies were computed. The statistical significance of relationships between variables was determined using Pearson’s Chi-squared test.

## Results

The participants in this study were 15 street vendors selling groundnuts, nabag, and tasali. The population was divided into six age groups: 10–20, 21–30, 31–40, 41–50, 51–60, and over 61 years. The most common age group was 31–40 (46.7%), followed by 41–50 (20%), 21–30 (13.3%), 10–20 (6.7%), 51–60 (6.7%), and more than 61 (6.7%). Eight (53.3%) of the 15 subjects were women, while seven (46.7%) were men.

The total parasitic contamination rate was 60% (27 samples), divided as follows: 20% (n = 9) for groundnut, 22.2 % (n = 10) for nabag, and 17.8 % (n = 8) for tasali. A Chi-squared test was used to test the relationship between crop type and detection of parasites, revealing an insignificant relationship with *P* = 0.757 ( Table 1). The presence of both *E. histolytica*/ *E.dispar*/ *E. moshkovskii* and *G. lamblia* accounted for 11.1% of the positive results.

**Table 1.** Frequency of study subjects according to age groups.

Age groups (years)	Frequency		Total
	Males	Females	
10–20	1	0	1 (6.7%)
20–30	1	1	2 (13.3%)
30–40	3	4	7 (46.7%)
40–50	2	1	3 (20%)
50–60	0	1	1 (6.7%)
More than 61	0	1	1 (6.7%)
Total	7 (46.7%)	8 (53.3%)	15 (100%)
	15 (100%)		

The contamination rate assessed using FECT was 53.3% (24 samples), while the sugar floatation technique detected a contamination rate of 48.9% (22 samples); the correlation between detection and technique used was found to be highly significant at *P* = 0.000 ( Table 2). Using FECT, the detected prevalence of *E. histolytica*/ *E.dispar*/ *E. moshkovskii, G. lamblia*, and mixed contamination of *E. histolytica*/ *E.dispar*/ *E. moshkovskii* and *G. lamblia* were 33.3% (15), 11.1% (5), and 8.9% (4), respectively; when using the sugar floatation technique, the detected prevalence of *E. histolytica*/ *E.dispar*/ *E. moshkovskii, G. lamblia*, and mixed contamination of *E. histolytica*/ *E.dispar*/ *E. moshkovskii*and *G. lamblia* were 37.8% (17), 8.9% (4), and 2.2% (1), respectively ( Table 2). The prevalence rates of *E. histolytica*/ *E.dispar*/ *E. moshkovskii* in groundnuts, nabag, and tasali were 15.6% (7), 8.9% (4), and 8.9% (5), respectively, and for *G. lamblia* were 2.2% (1), 6.7% (3), and 6.7% (3) ( Table 3). Mixed contamination was found in groundnuts, nabag, and tasali in the following proportions: 2.2% (1), 6.7% (3), and 2.2% (1), respectively. Contamination rates for groundnut, nabag, and tasali detected by FECT were 20% (9), 20% (9), and 13.3% (6), respectively, while those detected using the sugar floatation technique were 20% (9), 13.3 % (6), and 15.5% (7).

**Table 2.** Comparison between parasite species detected and technique used.

Parasite spp.	Technique, % (n)			
	FECT		Sugar	
	Positive	Negative	Positive	Negative
*E. histolytica*/ *E.dispar*/ *E. moshkovskii*	33.3 (15)	0 (0)	37.8 (17)	0 (0)
*G. lamblia*	11.1 (5)	0 (0)	8.9 (4)	0 (0)
*E. histolytica*/ *E.dispar*/ *E. moshkovskii* and *G. lamblia*	8.9 (4)	0 (0)	2.2 (1)	0 (0)
None	0 (0)	46.7 (21)	0 (0)	51.1 (23)
**Total**	53.3 (24)	46.7 (21)	48.9 (22)	51.1 (23)
	100 (45)		100 (45)	

P=0.000

**Table 3.** Correlation between crop type and detected parasite species.

Crop type	Parasite spp., % (n)			Total
	*E. histolytica*/ *E.dispar*/ *E. moshkovskii*	*G. lamblia*	*E. histolytica*/ *E.dispar*/ *E. moshkovskii* & *G. lamblia*	
Groundnuts	15.6 (7)	2.2 (1)	2.2 (1)	20 (9)
Nabag	8.9 (4)	6.7 (3)	6.7 (3)	22.3 (10)
Tasali	8.9 (4)	6.7 (3)	2.2 (1)	17.8 (8)
Total	33.4 (15)	15.6 (7)	11.1 (5)	60.1 (27)

P=0.757

The relationship between crop type positivity to contamination and technique used was negligible for both FECT ( *P* = 0.655) and sugar floatation technique ( *P* = 0.591) ( Table 4). The 31-40 age group had the highest contamination rate (33.3%), followed by 41–50 (13.3%), more than 61+ (6.7%), 51–60 (4.4%), 21–30 (2.2%), and 10–20 (0%) groups. There was a significant association between seller age group and outcome positivity ( *P* = 0.028). Contamination rates were 24.4%, 20%, and 15.6% in Khartoum state cities, i.e. Khartoum, Bahri, and Omdurman, respectively. There was no relation between city and contamination rate ( *P* = 0.329). The results revealed that *E. histolytica*/ *E.dispar*/ *E. moshkovskii* was the dominant parasite across all cities, with prevalence rates of 17.8%, 17.8%, and 8.9 % in Khartoum, Bahri, and Omdurman, respectively; while *G. lamblia* had lower prevalence rates in Khartoum, Bahri, and Omdurman, at 13.4%, 4.4%, and 8.9%, respectively. Relationship testing between city and species detected yielded insignificant results ( *P* = 0.460).

**Table 4.** Comparison between contaminated crop type and technique used.

Crop type	Positive results, % (n)	
	FECT	Sugar floatation
Groundnut	20 (9)	20 (9)
Nabag	20 (9)	13.3 (6)
Tasali	13.3 (6)	15.5 (7)
Total	53.3 (24)	48.8 (22)

## Discussion

Groundnut and watermelon seeds are important cash crops. They respectively represented 43,532 USD (59,620 tons) and 49,355 USD (74,149 tons) of Sudanese exports in 2018 ^18^. To the best of our knowledge, this study was the first to look into the parasitic contamination of groundnuts, nabag, and tasali sold by Sudanese street vendors. Two studies in Ghana and Nigeria estimated parasitic infections in the root plant *Cyperus esculentus L.* (tiger nuts); those studies were similar to some degree, and found contamination to be significant ^6, 7^. The overall contamination rate in the present study was 60%, which is considered significant. This rate was expected, because vendors sold their products unprotected and handled them with their bare hands. Contamination does not necessarily happen at the selling stage; it can happen during crop farming, harvesting, storage and transport, and even at home, according to Idahosa, 2011 ^19^ and Porter *et al*., 1990 ^20^. Contamination may occur during the planting phase as a result of polluted irrigational water, as mentioned by Keraita *et al.,* 2002 ^21^, which is contaminated as a result of inadequate or insufficient sanitation infrastructures to cope with the rate of urbanization ^22^. In our study, the most contaminated crop was nabag (22.2%), followed by groundnut (20%), and finally tasali. This may be due nabag being sold raw, which also exposes it to the previously mentioned contamination factors, particularly during the growing and harvesting phases; When nuts fall to the ground, they may come into contact with potentially polluted soil, as well as other external contaminants carried by wind, humans, or animals.

However, because groundnuts grow beneath the soil surface ^13^, they might be damaged if the nuts comes into contact with soil that has been contaminated, or with polluted irrigational water.

Despite this, salting and roasting may help to reduce contamination. Tasali had the lowest contamination rate in comparison to others. This could be explained by it being protected by the fruit (watermelon) during growing and harvesting, as well as the washing, salting, and roasting processes that occur before it is eaten. In our study, the parasites detected belonged to two species: *E. histolytica*/ *E.dispar*/ *E. moshkovskii* (44.4%) and *G. lamblia* (26.7%). *E. histolytica*/ *E.dispar*/ *E. moshkovskii* was the most common of both in all sampled crops (groundnut: 17.8% *vs* 4.4%, nabag: 15.6% *vs* 15.6%, and tasali: 11.1% *vs* 8.9%). Groundnuts had the highest rate of *E. histolytica*/ *E.dispar*/ *E. moshkovskii* contamination (44.4%). This finding is consistent with a Nigerian study on tiger nuts, which are similar to groundnuts in their cultivation process, and in which *E. histolytica*/ *E.dispar*/ *E. moshkovskii* was identified as the only protozoan parasite (25% contamination rate) ^6, 7, 13^. The precise prevalence rates of *E. histolytica*/ *E.dispar*/ *E. moshkovskii* and *G. lamblia* in Khartoum State are unknown; however, some studies may help to provide a more concise view *. E. histolytica*/ *E.dispar*/ *E. moshkovskii* and *G. lamblia* were found to be common parasites in the following areas: Alhag-yousif ( *G. lamblia*: 46.4% and *E. histolytica*/ *E.dispar*/ *E. moshkovskii*: 15.50%), Elengaz ( *G. lamblia*: 33.4% and *E. histolytica*/ *E.dispar*/ *E. moshkovskii*: 3.6%), and Alkalakla ( *G. lamblia*: 33.4% and *E. histolytica*/ *E.dispar*/ *E. moshkovskii*: 3.6%) ^17, 23, 24^. These results suggest that contaminated crop products could be a major source of infection.

Between the saturated sugar floatation technique (prevalence rate: 48.9%) and FECT (prevalence rate: 53.3%), the FECT was the found the most efficient technique for crop examination ( *P* = 0.000). This finding was supported by Abdalazim *et al.,* (2019) ^17^ ( *P* = 0.000). FECT outperformed the saturated sugar floatation technique in detecting both parasites, with FECT detecting 44.2% of *E. histolytica*/ *E.dispar*/ *E. moshkovskii* and *G. lamblia* while the sugar floatation technique detected 40% of *E. histolytica*/ *E.dispar*/ *E. moshkovskii* and 11.1% of *G. lamblia.* According to our study, seller sex and location (stationed vehicle or no vehicle) did not affect the positivity of crop samples to contamination (P-value: 0.807 and 0.329 respectively), while the age group played a role in the occurrence of contamination ^25^.

## Conclusions

Roasted groundnut, nabag, and tasali seeds sold by Sudanese street sellers were tainted with protozoan parasites. In all seeds examined, *E. histolytica/ E.dispar/ E. moshkovskii* was the dominant parasite. Although female vendors had a greater prevalence of contamination, the connection between seller sex and positivity to contamination was not significant.

Expanding the quantity of soil samples gathered and examined from farmers, central markets, and street vendors. Increasing the sample size and include additional goods from the vendors. Attempting to scan the soil in crop-growing areas and compare the results to those from central markets in order to pinpoint when contamination happened.

## Data availability

### Underlying data

Figshare: Parasitic Contamination Rate of Arachishypogaea L (Groundnuts), Citrulluslanatus Seeds (Watermelon Seeds), and Ziziphusspina-christi (Nabag) Sold by Street Venders in Khartoum State- Sudan. https://doi.org/10.6084/m9.figshare.14397914.v4 ^25^.

Data are available under the terms of the Creative Commons Attribution 4.0 International license (CC-BY 4.0).
