# Supplementary material for: Frequency of parasitic infections in Arachishypogaea L (groundnuts), Citrulluslanatus seeds (watermelon seeds), and Ziziphusspina-christi (nabag) sold by street vendors in Khartoum State, Sudan: a cross-sectional study
Source: F1000Res. 2021 Dec 15;10:586. Originally published 2021 Jul 16. [Version 3] doi: 10.12688/f1000research.53682.3 (PMC8886169; doi:10.12688/f1000research.53682.3)
Supplement: Peer review history

# Peer review: Reviewer response for version 3

**DOI:** 10.5256/f1000research.80827.r115327

**Published:** 2021-12-22

**Authors:** Samar Al Nahhas

**Affiliations:** 1 Department of Animal Biology, Faculty of Science, Damascus University, Damascus, Syria

After reviewing the revised manuscript, I found it OK. I approve it to be indexed.

Is the work clearly and accurately presented and does it cite the current literature?

Yes

If applicable, is the statistical analysis and its interpretation appropriate?

Yes

Are all the source data underlying the results available to ensure full reproducibility?

Yes

Is the study design appropriate and is the work technically sound?

Yes

Are the conclusions drawn adequately supported by the results?

Yes

Are sufficient details of methods and analysis provided to allow replication by others?

Yes

Reviewer Expertise:

Parasitology, infectious diseases, molecular diagnosis, immuno diagnosis, microbiology, enteric parasites, leishmaniasis, malaria, toxoplasmosis, echinococcusis.

I confirm that I have read this submission and believe that I have an appropriate level of expertise to confirm that it is of an acceptable scientific standard.

# Peer review: Reviewer response for version 3

**DOI:** 10.5256/f1000research.80827.r115326

**Published:** 2021-12-21

**Authors:** Erastus Mulinge

**Affiliations:** 1 Centre for Microbiology Research, Kenya Medical Research Institute, Nairobi, Kenya

The authors did not revise the conclusion of the manuscript as suggested, as per my previous comments (“The conclusion seems to be a continuation of the results section. Please make conclusions based on these findings, like the implication of these findings to public health and possible control measures as well as what further studies could focus on.”) or given reasons for not revising it.

Is the work clearly and accurately presented and does it cite the current literature?

Yes

If applicable, is the statistical analysis and its interpretation appropriate?

Yes

Are all the source data underlying the results available to ensure full reproducibility?

Yes

Is the study design appropriate and is the work technically sound?

Yes

Are the conclusions drawn adequately supported by the results?

No

Are sufficient details of methods and analysis provided to allow replication by others?

Yes

Reviewer Expertise:

Molecular epidemiology, Parasitology and One Health

I confirm that I have read this submission and believe that I have an appropriate level of expertise to confirm that it is of an acceptable scientific standard, however I have significant reservations, as outlined above.

# Peer review: Reviewer response for version 2

**DOI:** 10.5256/f1000research.79668.r101448

**Published:** 2021-12-09

**Authors:** Erastus Mulinge

**Affiliations:** 1 Centre for Microbiology Research, Kenya Medical Research Institute, Nairobi, Kenya

The authors revised the manuscript according to the comments I raised besides the major issue on the conclusion.

Is the work clearly and accurately presented and does it cite the current literature?

Yes

If applicable, is the statistical analysis and its interpretation appropriate?

Yes

Are all the source data underlying the results available to ensure full reproducibility?

Yes

Is the study design appropriate and is the work technically sound?

Yes

Are the conclusions drawn adequately supported by the results?

No

Are sufficient details of methods and analysis provided to allow replication by others?

Yes

Reviewer Expertise:

Molecular epidemiology, Parasitology and One Health

I confirm that I have read this submission and believe that I have an appropriate level of expertise to confirm that it is of an acceptable scientific standard, however I have significant reservations, as outlined above.

# Peer review: Reviewer response for version 2

**DOI:** 10.5256/f1000research.79668.r101449

**Published:** 2021-11-30

**Authors:** Samar Al Nahhas

**Affiliations:** 1 Department of Animal Biology, Faculty of Science, Damascus University, Damascus, Syria

After reviewing the revised paper, the authors did the requested changes.

Is the work clearly and accurately presented and does it cite the current literature?

Yes

If applicable, is the statistical analysis and its interpretation appropriate?

Yes

Are all the source data underlying the results available to ensure full reproducibility?

Yes

Is the study design appropriate and is the work technically sound?

Yes

Are the conclusions drawn adequately supported by the results?

Yes

Are sufficient details of methods and analysis provided to allow replication by others?

Yes

Reviewer Expertise:

Parasitology, infectious diseases, molecular diagnosis, immuno diagnosis, microbiology, enteric parasites, leishmaniasis, malaria, toxoplasmosis, echinococcusis.

I confirm that I have read this submission and believe that I have an appropriate level of expertise to confirm that it is of an acceptable scientific standard.

# Peer review: Reviewer response for version 1

**DOI:** 10.5256/f1000research.57096.r98829

**Published:** 2021-11-19

**Authors:** Erastus Mulinge

**Affiliations:** 1 Centre for Microbiology Research, Kenya Medical Research Institute, Nairobi, Kenya

The manuscript describes intestinal parasite contamination in food items sold by street vendors in Khartoum, Sudan. These parasitic agents detected are significant causes of diarrhea and other gastrointestinal symptoms which are major public health problems and therefore this manuscript deserves indexing.

**Major issues**

1. The conclusion seems to be a continuation of the results section. Please make conclusions based on these findings, like the implication of these findings to public health and possible control measures as well as what further studies could focus on.
2. Clarify the difference: the text (page 4) stated that 21 samples were positive for parasites by sugar flotation but in the table, it is 22.

**Minor issues**

1. Replace *Entamoeba histolytica,* address this as *Entamoeba histolytica*/ *E.dispar*/ *E. moshkovskii* throughout the manuscript.
2. Look carefully throughout the manuscript and clear all the spacing mistakes.
3. Change the last sentence in page 3 to *G. lamblia, E. histolytica, A. lumbricoides, Trichuris trichiura, A. doudenale, N. americanus *and *S. stercoralis.*
4. Avoid the word "then" in the methods.
5. Show p values for correlations in tables 2 and 3.
6. Results: the last paragraph, "The results revealed that *E. histolytica* was the dominant parasite across all city, with prevalence rates" – change to "all cities".
7. Discussion: the previous study was 60% - do you mean the present study?

Is the work clearly and accurately presented and does it cite the current literature?

Yes

If applicable, is the statistical analysis and its interpretation appropriate?

Yes

Are all the source data underlying the results available to ensure full reproducibility?

Yes

Is the study design appropriate and is the work technically sound?

Yes

Are the conclusions drawn adequately supported by the results?

No

Are sufficient details of methods and analysis provided to allow replication by others?

Yes

Reviewer Expertise:

Molecular epidemiology, Parasitology and One Health

I confirm that I have read this submission and believe that I have an appropriate level of expertise to confirm that it is of an acceptable scientific standard, however I have significant reservations, as outlined above.

# Response

**Published:** 2021-11-20

**Authors:** Ahmed Abd Alla

**Affiliations:** Sudan University of Science and Technology, Sudan

Major issues

- The conclusion seems to be a continuation of the results section. Please make conclusions based on these findings, like the implication of these findings to public health and possible control measures as well as what further studies could focus on.

**Author response:** I added a paragraph in the conclusion section in response to this. 

- Clarify the difference: the text (page 4) stated that 21 samples were positive for parasites by sugar flotation but in the table, it is 22.

**Author response:** I revised my data and I found the table is right, so I changed it in text. 

 Minor issues

- Replace *Entamoeba histolytica,* address this as *Entamoeba histolytica*/ *E.dispar*/ *E. moshkovskii* throughout the manuscript.

**Author response:** I respond to these all throughout the text and I changed all *E. histolytica* with *histolytica*/ *E.dispar*/ *E. moshkovskii.*

- Look carefully throughout the manuscript and clear all the spacing mistakes.

**Author response:** I respond to this. 

- Change the last sentence in page 3 to *G. lamblia, E. histolytica, A. lumbricoides, Trichuris trichiura, A. doudenale, N. americanus *and *S. stercoralis.*

**Author response:** I respond *.*

- Avoid the word "then" in the methods.

**Author response: **I respond to this.

- Show p values for correlations in tables 2 and 3.

**Author response:** l added p-value under Tables 2 and 3.

- Results: the last paragraph, "The results revealed that *E. histolytica* was the dominant parasite across all city, with prevalence rates" – change to "all cities".

**Author response: **I changed it as the reviewer said. 

- Discussion: the previous study was 60% - do you mean the present study?

**Author response:** I changed 'previous' to 'present'.

 I hope I clarify all the things.

# Peer review: Reviewer response for version 1

**DOI:** 10.5256/f1000research.57096.r98828

**Published:** 2021-11-15

**Authors:** Samar Al Nahhas

**Affiliations:** 1 Department of Animal Biology, Faculty of Science, Damascus University, Damascus, Syria

This scientific article addresses the frequency of parasitic infections in some vegetative crops. It addresses an important topic concerning both human and animal nutrition. Raw vegetables and fruits are one of the most essential sources of nourishment.  They often contribute to an epidemiological role in the transmission of parasitic food-borne diseases. Usually, fruits and vegetables become contaminated with different parasitic phases (cyst, oocyst, ova, and larvae) during irrigation by waste water or by direct contamination from animals and humans during harvesting, packing, transport processing, distribution, and marketing.

 In Sudan as in many countries revealed the presence of different parasites species like soil worms, protozoa, fungus…etc.

 Identifying parasitic contamination will help fight these infections, because knowing contamination rates allows applying strict control of fruit and vegetable cultivation areas, such as keeping animals far from direct contact with agricultural crops, periodic inspection of water sources used in irrigation, as well taking the necessary preventive measures.

 I have some points that need to be clarified:

- The authors record the Sellers ‘gender and age group, **what is the benefit**?  Page 5: last paragraph before discussion, they mentioned “The 31-40 age group had the highest contamination rate …… outcome positivity”. **Have the authors tested stool samples isolated from vendors**?
- It is not possible to distinguish and differentiate *E. histolytica* from the morphologically identical nonpathogenic species *E. dispar* and *E. moshkovskii* using light microscopy. **How did the authors confirm the presence of *Entamoeba histolytica*?**
- **The authors applied two techniques: FECT and sugar floatation. **What is the benefit? ****

Is the work clearly and accurately presented and does it cite the current literature?

Yes

If applicable, is the statistical analysis and its interpretation appropriate?

Yes

Are all the source data underlying the results available to ensure full reproducibility?

Yes

Is the study design appropriate and is the work technically sound?

Yes

Are the conclusions drawn adequately supported by the results?

Yes

Are sufficient details of methods and analysis provided to allow replication by others?

Yes

Reviewer Expertise:

parasitology, infectious diseases, molecular diagnosis, immuno diagnosis, microbiology, enteric parasites, leishmaniasis, malaria, toxoplasmosis, echinococcusis....

I confirm that I have read this submission and believe that I have an appropriate level of expertise to confirm that it is of an acceptable scientific standard, however I have significant reservations, as outlined above.

# Response

**Published:** 2021-11-20

**Authors:** Ahmed Abd Alla

**Affiliations:** Sudan University of Science and Technology, Sudan

1. Here we need to know the effect of gender and age group in the transmission of parasites through the crops, although some crops are sold by females more than males, like tasali.
2. We didn't take stool specimens, but of course, we mean by positive the crops are affected not the person.
3. Yes, it's difficult to distinguish between the species by light microscope, so we change all *E. histolytica* to *E. histolytica/E.dispar/E.moshkovskii.*
4. Here we want to compare which is the best.
